# Effects of exercise training on the nigrostriatal glutamatergic pathway and receptor interactions in Parkinson’s disease: a systematic review

**DOI:** 10.3389/fnagi.2025.1512278

**Published:** 2025-02-11

**Authors:** Shahid Ishaq, Iqbal Ali Shah, Shin-Da Lee, Bor-Tsang Wu

**Affiliations:** ^1^PhD Program in Healthcare Science, College of Healthcare Science, China Medical University, Taichung, Taiwan; ^2^Department of Physical Therapy, China Medical University, Taichung, Taiwan; ^3^Department of Senior Citizen Service Management, National Taichung University of Science and Technology, Taichung, Taiwan

**Keywords:** glutamate, glutamatergic system, glutamate receptors, nigrostriatum, Parkinson’s disease, Parkinson, metabotropic receptors

## Abstract

**Background:**

The excitatory imbalance of glutamatergic neurons, caused by insufficient input from dopaminergic neurons, contributes the pathogenesis of Parkinson’s disease (PD). Exercise training is one of the non-pharmacological, non-invasive therapeutic approaches.

**Objective:**

This systematic review is the first to summarize the effects of exercise training on the regulation of protein and gene expressions within the nigrostriatal glutamatergic pathway and its receptor interactions in animal models of Parkinson’s disease (PD).

**Methodology:**

The PubMed, Web of Science, and Embase electronic databases were searched, and 9 out of 96 studies that met the PRISMA guidelines were included. These studies received a CAMARADES score ranging from 4 to 6 out of 10. The included studies utilized pharmacologically induced PD models in mice or rats with 1-methyl-4-phenyl-1,2,3,6-tetrahydropyridine (MPTP) or 6-hydroxydopamine (6-OHDA). The majority of studies (89%) employed treadmill training, while 11% used voluntary wheel running, with training protocols consisting of 5 days per week for 4 weeks.

**Results:**

Exercise training reduced extracellular glutamate (Glu) and increased the expression of GLT-1, GS, Gln, and mGluR2/3 while down-regulating VGULT1 in the presynaptic terminal of the glutamatergic neurons within the nigrostriatal pathway in PD animal models. It also downregulated mGluR5 and modulated the *α*-amino-3-hydroxy-5-methyl-4-isoxazolepropionic acid (AMPA) receptor subunits: GluA1 was downregulated, inhibiting long-term potentiation, while GluA2 and GluA3 were upregulated in the nigrostriatal pathway in PD animal models. In addition, the exercise training downregulated the N-methyl-D-aspartate (NMDA) receptors, Arc, Cav1.3, CaMKII, and p-CaMKII in the nigrostriatal pathway in PD animal models.

**Conclusion:**

Exercise training exerted a neuroprotective effect on the glutamatergic pathway in Parkinson’s disease (PD) animal models by limiting excess glutamate in the synaptic cleft. Exercise training modulated the ionotropic receptors and limited the glutamatergic excitatory imbalance within the nigrostriatal pathway in PD animal models. It also improved motor function, including balance, coordination, and gait parameters.

**Systematic review registration:**

https://www.crd.york.ac.uk/prospero/#recordDetails, identifier CRD42024564127

## Introduction

Parkinson’s disease (PD) is a degenerative disease of the central nervous system (CNS) characterized by progressive motor dysfunction. This condition results from insufficient activation of the nigrostriatal dopaminergic pathway and overactivation of the corticostriatal glutamatergic pathway, leading to abnormal excitatory postsynaptic potentials (EPSPs) (Zhao et al., 2022; Hou et al., 2017). In PD, the degeneration of dopaminergic neurons in the substantia nigra disrupts dopamine-mediated regulation of glutamate release from corticostriatal neurons, causing excessive glutamate release and overactivation of N-methyl-D-aspartate (NMDA) and *α*-amino-3-hydroxy-5-methyl-4-isoxazolepropionic acid (AMPA) receptors, which lead to neurodegeneration and motor dysfunction (Wang et al., 2022; Dong et al., 2009; Meredith et al., 2009). Current treatments for neurodegenerative diseases often have side effects, while exercise training offers a non-invasive, non-pharmacological alternative that has been shown to alleviate motor and cognitive symptoms without adverse effects. It may also prevent extracellular concentrations of glutamate (Glu) and preserve glutamatergic excitatory imbalance, thereby exerting neuroprotective effects on nigrostriatal neurons in PD (Shi et al., 2019; Petzinger et al., 2011). Furthermore, it has been reported that exercise training improves PD symptoms, balance, and independence in daily activities, along with enhancing walking abilities such as walking speed, stride length, stride frequency, and total walking distance (Palasz et al., 2019; Goodwin et al., 2008; Chen et al., 2018).

In glutamatergic regulation, glutamate (Glu), the most abundant neurotransmitter in the central nervous system, acts on ionotropic receptors such as N-methyl-D-aspartate (NMDA), *α*-amino-3-hydroxy-5-methyl-4-isoxazolepropionic acid (AMPA), and kainate (KA) and also on metabotropic G protein-coupled receptors, all of which are crucial for normal brain function. In contrast, glutamatergic overactivation triggers neuronal damage in PD (Dietrich et al., 2005; Garcia et al., 2010). Neurodegeneration in PD animals increases the density of glutamatergic immunolabeling in nerve terminals of striatal medium spiny neurons (MSNs) (Fisher et al., 2004). AMPA receptors mainly manage Na^+^ flux and some Ca^2+^, while NMDA receptors mainly manage large quantities of Ca^2+^. Glutamate-induced elevation of cytosolic Ca^2+^ levels activates protein kinases, such as calcium/calmodulin-dependent protein kinase II (CaMKII) and Cav1.3, which is abundant in medium spiny neurons of the striatum. This elevation also triggers transcription factors such as cyclic AMP response element-binding protein (CREB), leading to increased glutamatergic hyperactivity and neurodegeneration in PD animal models (Chen et al., 2017; Mattson, 2008; Garcia et al., 2010). Metabotropic glutamate receptors (mGluRs), particularly striatal mGluR5 and mGluR2/3, are promising targets for PD treatment due to their roles in modulating presynaptic glutamate release, intracellular Ca^2+^ levels, and neuroprotection. Interventions targeting these receptors have shown potential in alleviating PD symptoms and reducing dendritic spine loss in the striatum of PD animal models (Shi et al., 2019; Garcia et al., 2010).

Damage to dopaminergic neurons in the substantia nigra reduces dopamine levels in the striatum, which, in turn, leads to overactivation of the glutamatergic pathway, causing an excitatory imbalance that exacerbates neural dysfunction (Chen et al., 2017). The glutamate transporter GLT-1, the ion channel affinity protein GLAST, and EAAC1 play a crucial role in maintaining homeostasis by removing excess Glu from the synaptic cleft in the striatum of PD animal models (Feng et al., 2021; Arnold and Salvatore, 2016).

Exercise training exerts neuroprotective effects by modulating the molecular mechanism and is beneficial for symptomatic recovery. The literature supports the effects of exercise training on motor recovery but presents conflicting evidence regarding the recovery of the glutamatergic pathway within the nigrostriatal pathway in animals with PD (Sconce et al., 2015). This systematic review is the first to summarize the effects of exercise training on regulating the glutamatergic pathway and its receptor interactions in the nigrostriatal pathway in animal models of Parkinson’s disease (PD).

## Methodology

### Study design

This systematic review included randomized controlled trials and controlled trials with separate exercise training and well-defined control groups. Only full-text articles published in English with detailed descriptions of PD animal models, sample sizes, types of exercise training, and relevant outcome measures were included in this study. Studies that included an exercise group combined with any other parallel interventions or those that lacked a non-exercise control group were excluded from the current systematic review. Additionally, studies that did not implement an exercise training program for at least 4 weeks were also excluded. Cross-over studies, review articles, case reports, and conference papers were also excluded from this study.

### Search strategies

PubMed, Embase, and the Web of Science were searched comprehensively using the following search string: “(treadmill training OR exercise training OR physical training) AND (Parkinson’s OR Parkinson’s Disease) AND (Glutamate OR Glutamatergic system OR Glutamate receptors) AND animal.” After removing duplicate results, two independent reviewers screened the titles and abstracts for eligibility, followed by a full-text screening of eligible studies. Tables, figures, graphs, and text were reviewed to extract relevant outcome data.

### Outcome measures

This study summarized proteins and gene expressions involved in the glutamatergic pathway within the nigrostriatal pathway and regulated by exercise training as primary outcome measures. Protein and gene expressions or molecular mechanisms involving any brain regions or pathways other than our target locations or pathways were excluded from this research. This study summarized motor behavior, including walking, balance, and gait, as secondary outcome measures. This review included only published articles and excluded those in the process of publication. A senior specialist in the field was consulted to resolve any ambiguities in the process.

### Data extraction

Two reviewers independently extracted data from the eligible studies, including study characteristics such as author names, year of publication, sample size, gender, age, and weight. Details of disease models were collected, including the methods of disease induction, whether genetic or pharmacological, such as MPTP or 6-OHDA. This study primarily focused on the brain regions of the substantia nigra and striatum. The types of exercise, including treadmill training and voluntary wheel running, were documented, along with the exercise parameters, such as speed, session duration, and frequency of 3–5 days per week for at least 4 weeks. Motor function measurements were extracted along with the glutamatergic pathway outcomes. Results of western blotting, ELISA, and immunofluorescence were recorded for protein expression, while real-time polymerase chain reaction (RT-PCR) and quantitative PCR (qPCR) results were extracted for gene expression.

### Quality assessment

Two reviewers independently assessed the quality of the included studies using the CAMARADES checklist. The checklist consists of 10 items that evaluate the key methodological quality of preclinical studies. Each study was evaluated based on these criteria, with one point assigned for each adequately reported item, up to a maximum score of 10. The final quality assessment scores were used to identify potential bias and to assess the overall reliability of the included studies. These scores were also considered in the data synthesis and interpretation of the findings.

## Results

**Figure 1:**
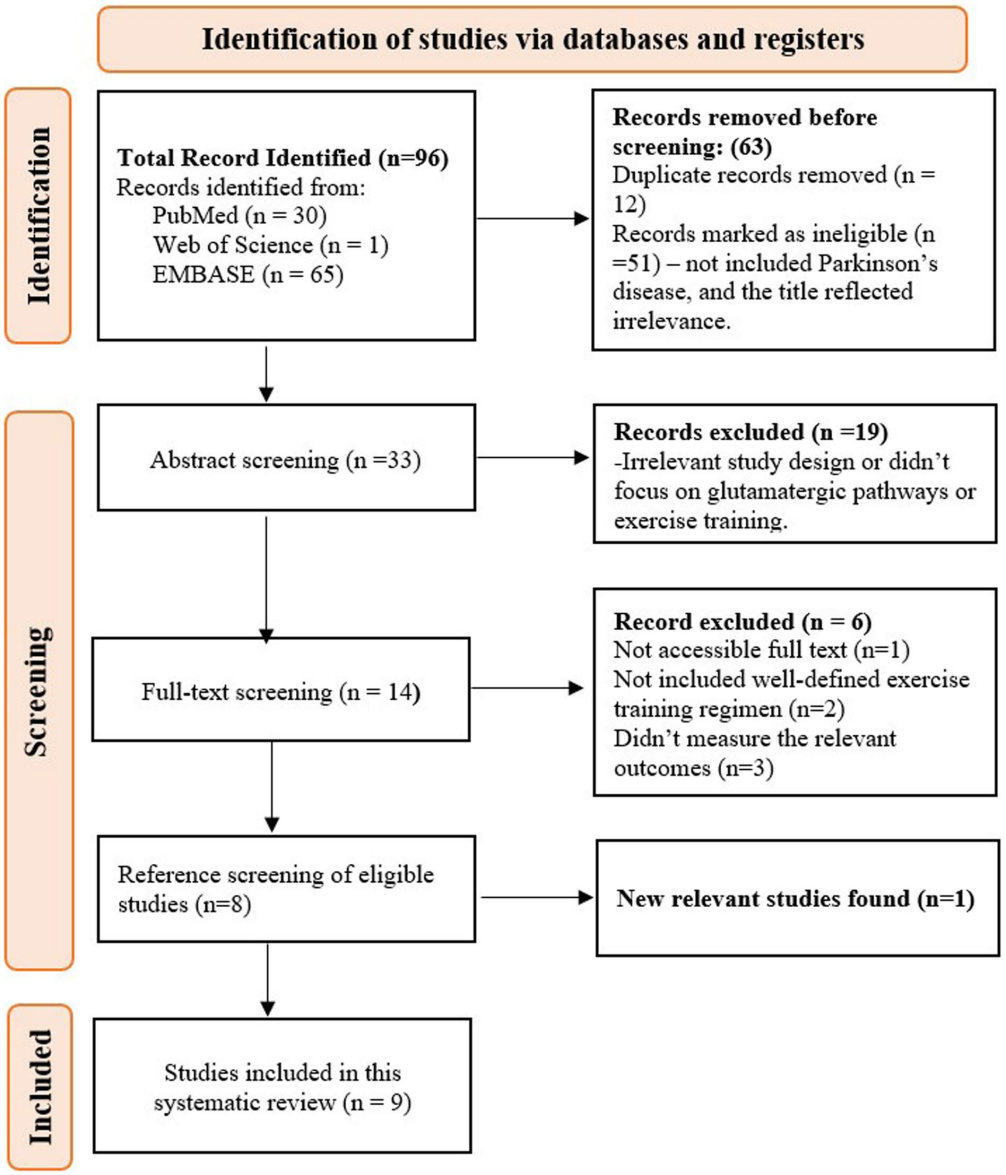
PRISMA flow diagram for study selection methodology.

The search yielded a total of 96 articles from PubMed (30), the Web of Science (1), and Embase (65). After removing 12 duplicates, 51 ineligible studies—those not related to Parkinson’s disease or with irrelevant titles—were excluded during the title screening. The abstracts of 33 studies were then screened, and 19 studies were removed due to irrelevant study designs or a lack of focus on glutamatergic pathways and exercise training. A total of 14 studies underwent full-text screening, with 6 studies excluded for the following reasons: full text not accessible (*n* = 1), lack of a well-defined exercise training regimen (*n* = 2), and failure to measure relevant outcomes (*n* = 3). Eight studies met the criteria and were included. After screening the references of these eight studies, one additional article was retrieved, bringing the total to nine studies (Figure 1).

All studies included rodent models with male animals aged between 6 and 12 weeks. The studies employed pharmacologically induced PD models, with 55.6% using the 6-OHDA model in rats and 45.4% using the MPTP model in mice. Regarding the exercise interventions, 89% of the studies implemented treadmill training, while 11% employed voluntary wheel running. The treadmill training regimen typically involved running at a speed greater than 10 m per min for 30–60 min per session, 3–5 days per week, for 4 weeks (Table 1).

The regulation of protein and gene expression involved in the nigrostriatal glutamatergic pathway and motor function following exercise training is summarized in Table 1. Motor function assessments revealed that the Parkinson’s disease (PD) group exhibited impairments such as decreased latency and total time on the balance bar, reduced coordination on the suspension test, and poorer performance on the body swing test. In contrast, the PD + Exercise (Ex) group showed improvements in these metrics, with increased latency and total time on the balance bar, enhanced coordination on the suspension test, and better performance on the body swing test (Feng et al., 2021). In addition, the PD group showed reduced total distance traveled, walking time, and velocity, whereas these metrics were significantly improved in the PD + Ex group (Shi et al., 2019; Sconce et al., 2015; VanLeeuwen et al., 2010). In the open field test (OFT), the PD group exhibited decreased total distance traveled and walking time, both of which improved following exercise training (Chen and Li, 2019). The cylinder test indicated that exercise training reduced walking asymmetry in the PD + Ex group compared to the PD group (Chen et al., 2017). Furthermore, the PD + Ex group showed increased stride frequency and stride length, both of which were found to be reduced in the PD group (Churchill et al., 2017).

**Table 1:** Summary of the effects of exercise training on the nigrostriatal glutamatergic pathway.

Author name	Sample	Disease model	Brain tissue	Exercise type	Exercise parameters	Motor function	Nigrostriatal glutamatergic regulation
Feng et al. (2021)	S: Male rats (*n* = 68). A: 7 weeks W: 240 ± 10 g	6-OHDA	SN, striatum	Treadmill training	Sp: 11 m/min, D: 30 min/d, F: 5 d/week P: 4 weeks	**PD:** latency and total time on crossing balance beam ↓, coordination on suspension test ↓, body swing test ↓ **PD + Ex:** latency and total time on crossing balance beam ↑, coordination on suspension test ↑, body swing test ↑	**PD:** Glu↑, GLT-1↓, mRNA-GLT1 ↓, GS ↓, mRNA-GS↓ **PD + Ex:** Glu↓, GLT-1↑, mRNA-GLT1↑ GS ↑, mRNA-GS↑
Chen and Li (2019)	S: Male rats (*n* = 36). A: 6 weeks W: 240 ± 10 g	6-OHDA	SN, striatum	Treadmill training	Sp: 11 m/min, D: 30 min/d, F: 5 d/week P: 4 weeks	**PD:** total distance traveled ↓, Walking time ↓, Velocity ↓ **PD + Ex:** total distance traveled ↑, Walking time ↑, Velocity ↑ (OFT)	**PD:** Glu↑, mGluR2↓, mRNA-mGluR2↓, mGluR3 ↓, mRNA-mGluR3↓, **PD + Ex:** Glu↓, mGluR2↑, mRNA-mGluR2Ө, mGluR3 ↑, mRNA-mGluR3↑
Shi et al. (2019)	S: Male rats (*n* = 72). A: 8 weeks W: 220–250 g	6 OHDA	Striatum	Treadmill training	Sp: 11 m/min, D: 30 min/d, F: 5 d/week P: 4 weeks	**PD:** total distance traveled ↓, Walking time ↓, **PD + Ex:** total distance traveled ↑, Walking time ↑, (Pan lab)	**PD:** Glu↑, mGluR2↓, mGluR3 ↓, mGluR5 ↑ **PD + Ex:** Glu↓, mGluR2↑, mGluR3 ↑, mGluR5 ↓
Chen et al. (2017)	S: Male rats (*n* = 280). A: 8 weeks W: 220−240 g	6 OHDA	Striatum	Treadmill training (Forced)	Sp: 11 m/min, D: 30 min/d, F: 7 d/week P: 4 weeks	**PD:** Walking asymmetry ↑, **PD + Ex:** Walking Asymmetry↓ (cylinder Test)	**PD:** Cav1.3↑ CaMKII↑, and p-CaMKII ↑(Thr286), NMDAR↑ **PD + Ex:** Cav1.3 ↓, CAMKII↓, p-CaMKII (Thr286)↓, NMDAR↓
Garcia et al. (2017)	S: Male rats (*n* =?). A: 12 weeks W: 280 − 300 g	6 OHDA	Striatum midbrain	Treadmill training	Sp: 10 m/min, D: 40 min/d, F: 3 d/week P: 4–16 weeks		**PD:** GluA1↑, GluA2↓, GluA3↓, Arc↑, **PD + Ex:** **4 weeks** GluA1↓, GluA2↑, GluA3 ↑, Arc↓
Churchill et al. (2017)	S: Male mice (*n* = 69). A: 8 weeks	MPTP	SN, striatum	Treadmill	Sp: 11 m/min, D: 60 min/d, F: 5 d/week P: 4 weeks	**PD:** Stride frequency ↓, stride length ↓, **PD + Ex:** stride frequency↑, Stride length ↑	**PD:** GLT-1↓, **PD + Ex:** GLT-1↑
Sconce et al. (2015)	S: Male mice (*n* = 31). A: 8 weeks W: 280 − 300 g	MPTP	SN, striatum	Voluntary wheel running	4 weeks	**PD:** total distance traveled ↓, Walking time ↓, **PD + Ex:** total distance traveled ↑, Walking time ↑	**PD:** GLT-1↑, VGULT1↑, EAAC1↑, GLAST↑ **PD + Ex:** GLT-1↓, VGULT1↓, EAAC1↓, GLAST↓
Kintz et al. (2013)	S: Male mice (*n* = 100). A: 8-10 weeks	MPTP	SN, Striatum	Treadmill Exercise	Sp: Prog. 10–24 m/min, F: 5 days/week P: 4 weeks		**PD:** GluA1↓, GluA2↓, **PD + Ex:** GluA1↓, GluA2↑
VanLeeuwen et al. (2010)	S: Male mice (*n* = 100). A: 8-10 weeks	MPTP	Striatum, Basal Ganglia	Treadmill exercise	Sp: 10 m/min, D: 30 min/d, F: 5 d/week P: 4 weeks	**PD:** total distance traveled ↓, Walking time ↓, **PD + Ex:** total distance traveled ↑, Walking time ↑	**PD:** GluA1 ↓, GluA2↓ **PD + Ex:** GluA1 ↓, GluA2↑

This table summarizes the effect of the exercise training (Ex) on the nigrostriatal glutamatergic pathways in the various animal models of Parkinson’s disease (PD): 1-methyl-4-phenyl-1,2,3,6-tetrahydropyridine (MPTP) and 6-hydroxydopamine (6-OHDA). It includes details on the subject (S), sample size (n), age (A), weight (W), gram (g), and substantia nigra (SN). The Ex parameters are speed (Sp), meter/min (m/min), time duration (D), frequency (F), and period (P). Parkinson’s group (PD), Parkinson’s and Exercise group (PD + Ex), and open field test (OFT). The following symbols are used: total (t), phosphorylation (p), decreased/downregulation (↓), increased/upregulation (↑), and no significant effect (Ө). Key molecular markers include the following: glutamate (Glu), glutamate transporter 1 (GLT-1) and its mRNA, glutamine synthetase (GS) and its mRNA, metabotropic glutamate receptors 2 and 3 (mGluR2/3), metabotropic glutamate receptor 5 (mGluR5), voltage-gated calcium channel subunit alpha 1D (Cav1.3), and calcium/calmodulin-dependent protein kinase II (CaMKII), including its phosphorylated form at threonine 286 (p-CaMKII). Ionotropic glutamate receptors 1, 2, and 3 (GluA1, GluA2, and GluA3), which are the subunits of the AMPA receptor, show differential expression with disease progression and exercise. Other relevant markers include the following: activity-regulated cytoskeleton-associated protein (Arc), vesicular glutamate transporter 1 (VGULT1), excitatory amino acid transporter 1 (EAAC1), and glutamate aspartate transporter (GLAST).

In terms of nigrostriatal glutamatergic regulation, the extracellular Glu levels were elevated in the PD group but significantly reduced in the PD + Ex group (Chen and Li, 2019; Feng et al., 2021; Shi et al., 2019). The astrocytic proteins, including GLT-1 and GS, were reduced in the PD group, limiting the conversion of Glu to glutamine (Gln), whereas their expression was upregulated in the PD + Ex group, thereby increasing the Gln levels (Feng et al., 2021; Churchill et al., 2017; Sconce et al., 2015; VanLeeuwen et al., 2010). In the presynaptic terminals of the glutamatergic neurons, the PD group exhibited increased VGULT1 expression, which was reversed in the PD + Ex group (Sconce et al., 2015). The PD group showed decreased expression of mGluR2 and mGluR3, whereas the PD + Ex group showed increased expression of these proteins in the presynaptic terminals of the glutamatergic neurons, thereby limiting Glu release into the synaptic cleft (Chen and Li, 2019; Shi et al., 2019).

The PD group showed increased mGluR5 expression in the postsynaptic terminal, enhancing Glu influx into the postsynaptic membrane and causing excitotoxicity to the glutamatergic neurons, while the PD + Ex group showed decreased mGluR5 expression (Shi et al., 2019). The AMPA receptor subunit modulation also revealed key differences: while GluA1 expression increased and GluA2 and GluA3 decreased in the PD group, the PD + Ex group exhibited reduced expression of GluA1 and increased GluA2 and GluA3 (VanLeeuwen et al., 2010; Kintz et al., 2013; Garcia et al., 2017). Concurrently, NMDA receptors, Cav1.3, p-CaMKII (Thr286), and Arc, which were elevated in the PD group and contributed to hyperactivation and excitotoxicity, were reduced in the PD + Ex group (Chen et al., 2017; Garcia et al., 2010). However, one study presented contradictory findings, reporting increased expression of GLT-1, EAAC1, and GLAST in the PD group, with reductions observed in the PD + Ex group (VanLeeuwen et al., 2010).

The methodological quality of the included studies, as assessed using the CAMARADES checklist, ranged from 4/10 to 6/10. All studies used PD animal models, adhered to compliance standards, and were published in peer-reviewed journals. A total of five of the nine studies reported temperature control and randomization, while seven of the nine studies addressed conflicts of interest. However, none of the studies provided information on allocation concealment, blinded assessments, anesthetic compliance, or sample size calculations (Table 2).

**Table 2:** The CAMARADES checklist.

Study	1	2	3	4	5	6	7	8	9	10	Total score
Feng et al. (2021)	✓	✓	✓				✓		✓		5/10
Chen and Li (2019)	✓	✓	✓				✓		✓	✓	6/10
Shi et al. (2019)	✓	✓					✓		✓	✓	5/10
Chen et al. (2017)	✓	✓	✓				✓		✓	✓	6/10
Garcia et al. (2017)	✓						✓		✓	✓	4/10
Sconce et al. (2015)	✓	✓	✓				✓		✓		5/10
Churchill et al. (2017)	✓		✓				✓		✓	✓	5/10
Kintz et al. (2013)	✓						✓		✓	✓	4/10
VanLeeuwen et al. (2010)	✓						✓		✓	✓	4/10

(1) Publication in a peer-reviewed journal; (2) statement of temperature control; (3) randomization to treatment or control; (4) allocation concealment; (5) blinded assessment of outcomes; (6) avoidance of anesthetics with marked intrinsic properties; (7) use of animals with PD; (8) sample size calculation; (9) statement of compliance with regulatory requirements; (10) statement regarding possible conflict of interest.

## Discussion

**Figure 2:**
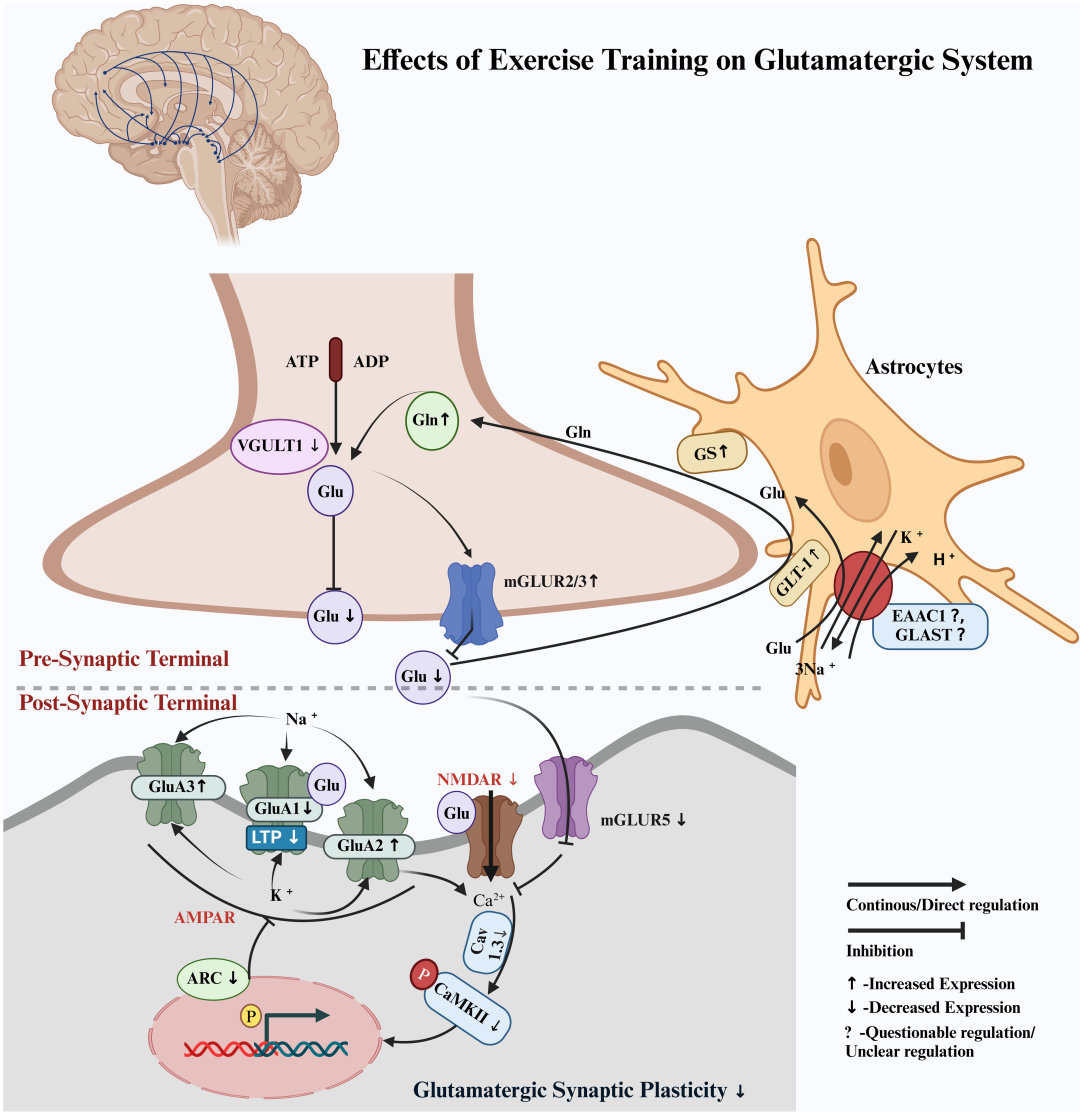
Hypothetical pathway illustrating the regulation of the glutamatergic system by exercise training. Pre-synaptic terminal: Glutamate (Glu), glutamate transporter 1 (GLT-1), glutamine synthetase (GS), glutamine (Gln), vesicular glutamate transporter 1 (VGULT1), and metabotropic glutamate receptors 2 and 3 (mGluR2/3). Post-synaptic terminal: metabotropic glutamate receptor 5 (mGluR5), voltage-gated calcium channel subunit alpha1D (Cav1.3), and calcium/calmodulin-dependent protein kinase II (CaMKII), including its phosphorylated form at threonine 286 (p-CaMKII). Ionotropic glutamate receptors 1, 2, and 3 (GluA1, GluA2, and GluA3), which are subunits of the AMPA receptor, show differential expression with disease progression and exercise. Activity-regulated cytoskeleton-associated protein (Arc), excitatory amino acid transporter 1 (EAAC1), glutamate aspartate transporter (GLAST), and phosphorylation (p).

Our findings demonstrated that exercise training exerted a neuroprotective effect on the glutamatergic pathway by modulating the activity of the glutamatergic neurons within the nigrostriatal pathway of Parkinson’s disease (PD) animal models, potentially reducing excitatory imbalances associated with dopaminergic depletion. Exercise training increased glutamate (Glu) reuptake from the synaptic cleft by enhancing the expression of the astrocyte protein glutamate transporter (GLT-1). This was followed by an increase in the expression of glutamate synthase (GS), which converted Glu to glutamine (Gln), subsequently releasing it into the extracellular space, where it was reabsorbed into the presynaptic terminal of glutamatergic neurons in the nigrostriatal pathway in PD animal models. Exercise training also limited Glu release into the synaptic cleft via VGULT1 and increased the expression of inhibitory mGluR2/3 receptors on the presynaptic membrane within the nigrostriatal pathway in PD animal models. It also reduced the excitotoxic effects on the glutamatergic neurons by decreasing the expression of the excitatory mGluR5 receptor in the nigrostriatal pathway in PD animal models. Exercise training effectively modulated ionotropic glutamate receptors, including AMPA (*α*-amino-3-hydroxy-5-methyl-4-isoxazolepropionic acid) subunits and NMDA (N-methyl-D-aspartate), which limited long-term potentiation and synaptic hyperactivity. It restored normal synaptic transmission in glutamatergic neurons within the nigrostriatal pathway in PD animal models (Figure 2). Exercise training increased PD latency; improved motor function, balance, and coordination; enhanced total distance, walking time, walking velocity, stride frequency, stride length, and reduced walking asymmetry in PD animal models compared to the non-exercised PD animal models. CAMARADES scores of 4–6/10 indicated moderate methodological quality in the included studies.

Our study consistently demonstrated that exercise training increased the reuptake of Glu from the synaptic cleft in the nigrostriatal region of the MPTP- and 6-OHDA-induced PD animal models and improved motor function (Feng et al., 2021; Chen and Li, 2019; Shi et al., 2019). Exercise training facilitated the reduction of extracellular Glu levels, limited abnormal burst firing, and restored normal synaptic transmission in the nigrostriatal glutamatergic pathway in PD animal models (Ferreira et al., 2021). A previous study reported that treadmill training for more than 4 weeks decreased the nerve terminal density of striatal medium spiny neurons, possibly reflecting a reduction in Glu levels in the synaptic cleft, and improved the running velocity of animals in MPTP-induced PD models (Fisher et al., 2004). Increased Glu levels in the extracellular space in PD lead to an increase in spontaneous excitatory postsynaptic currents (sEPSCs) in striatal medium spiny neurons (MSNs), causing hyperexcitation and damage to glutamatergic neurons. However, exercise training reduces sEPSCs in MSNs, protecting against damage and improving motor behavior (Zhao et al., 2022; Wang et al., 2022). Exercise training was found to improve the transmission of dopaminergic neurons and simultaneously normalize glutamate-driven spikes in the GABAA-mediated current through GABAA receptors and calcium channel blockers in the striatum, ameliorating spatiotemporal gait disturbances in the PD animal model (Chen et al., 2018; Hou et al., 2017).

Extracellular fluid in the central nervous system (CNS) does not contain Glu-metabolizing enzymes, so the removal of extracellular Glu relies primarily on inward uptake by transporters such as GLT-1, which terminates the synaptic effects of Glu and prevents the potential excitotoxic accumulation of extracellular Glu. Elimination of GLT-1 has been linked to dyskinesia and motor dysfunction in the striatum of animal models of PD (Farrand et al., 2016). In our study, exercise training facilitated the removal of Glu from the synaptic cleft by astrocytic GLT-1. After being converted to glutamine by the GS protein within the astrocytes, glutamine was subsequently transported to the presynaptic membrane. Along with the action of VGULT, this process limited Glu release into the synaptic cleft within the nigrostriatal pathway in PD animal models, thereby reducing MSN damage, exerting neuroprotective effects, and improving motor behavior (Feng et al., 2021; Churchill et al., 2017; Sconce et al., 2015).

According to our study findings, voluntary exercise training for 4 weeks downregulated the astroglial proteins EAAC1 and GLAST, which have an affinity for the Na + channels to reuptake Glu from the synaptic cleft within the nigrostriatal region of the MPTP-induced PD models. Although the decreased expression of these proteins raises questions about the neuroprotective effects, the study still demonstrated improvements in motor function (Sconce et al., 2015). In contrast, another study indicated that treadmill training for 4 weeks increased the expression of EAAC1 and GLAST in the substantia nigra, although not in the striatum, and improved motor function by increasing the total distance traveled and walking time in 18-month-old male Brown–Norway/Fischer 344 F1 hybrid rats (Arnold and Salvatore, 2016).

Our results also reported that 4 weeks of treadmill training significantly modulated the AMPA receptors. Specifically, the decreased expression of GluA1 limited long-term potentiation, while the increased expression of GluA2/3 enhanced ionic interactions with Na^+^ and Ca^2+^, impaired Mg^2+^ influx, and restored normal synaptic transmission in the nigrostriatal pathway in PD animal models. These molecular changes were associated with improved motor function, as reflected by increased total distance traveled, walking time, stride length, and stride frequency (VanLeeuwen et al., 2010; Kintz et al., 2013). However, one study reported similar results after 4 weeks of treadmill training, while conflicting results were observed after 16 weeks in the nigrostriatal region of PD models. The authors attributed this to the maintenance of homeostasis (Garcia et al., 2017). Another study supported the modulation of AMPA receptors following 4 weeks of voluntary exercise training but found no significant effects on NMDA receptors in the cortex of animals with neurodegenerative diseases (Dietrich et al., 2005).

Cav1.3 subunits, abundant in medium spiny neurons (MSNs), are crucial for excitation-transcription coupling with NMDA receptors in the striatum of animals with PD (Picconi et al., 2004). Upon Cav1.3 activation, intracellular Ca^2+^ levels increase, leading to auto-phosphorylation of Ca^2+^/calmodulin-dependent protein kinase II (CaMKII) at Thr286. This process is associated with hyperactivity in the corticostriatal glutamatergic pathway in the dopamine-depleted striatum (Mohanan et al., 2022). This study demonstrated that 4 weeks of treadmill training decreased the Cav1.3 subunits, inhibited NMDA receptors and auto-phosphorylation of CAMKII at Thr 286, decreased intracellular Ca2+ in the striatum, and improved walking asymmetry in the 6-OHDA-induced PD rat models (Chen et al., 2017).

## Limitations

This systematic review is limited by the relatively small number of studies that specifically examined the impact of exercise training on the glutamatergic pathway and its receptor interactions in animal models of Parkinson’s disease. The studies only included male animal models and primarily focused on gene and protein expressions within the nigrostriatal pathway, limiting the scope of the findings. The review did not address interactions with medium spiny GABAergic neurons. Due to a lack of relevant studies, it did not explore the glutamatergic pathway in regions such as the cortex-basal ganglia or other brain areas. In addition, this review did not include postsynaptic excitatory potentials, which could have provided a more comprehensive understanding of the glutamatergic system’s role in neuroprotection and motor function recovery. The included studies did not report on cognitive function in animals with PD.

## Conclusion

Exercise training modulated the glutamatergic pathway and exerted a neuroprotective effect in the nigrostriatal pathway in Parkinson’s disease (PD). It enhanced extracellular glutamate reuptake through astrocyte proteins such as GLT-1 and supported the conversion of glutamate to glutamine (Gln) via glutamine synthetase (GS). Exercise training also reduced glutamate release through VGluT1 and increased the expression of inhibitory mGluR2/3 receptors. In addition, at the postsynaptic terminal, it down-regulated excitatory mGluR5 receptors and modulated ionotropic AMPA and NMDA receptors, preventing calcium overload and abnormal glutamatergic excitation. Exercise training is a promising therapeutic approach for improving motor deficits and mitigating glutamatergic dysregulation in PD.

## Data Availability

The original contributions presented in the study are included in the article/supplementary material, further inquiries can be directed to the corresponding authors.
